# Vaccination coverage and factors influencing routine vaccination status in 12 high risk health zones in the Province of Kinshasa City, Democratic Republic of Congo (DRC), 2015

**DOI:** 10.11604/pamj.supp.2017.27.3.11930

**Published:** 2017-06-21

**Authors:** Guillaume Ngoie Mwamba, Norbert Yoloyolo, Yolande Masembe, Muriel Nzazi Nsambu, Cathy Nzuzi, Patrice Tshekoya, Barthelemy Dah, Guylain Kaya

**Affiliations:** 1 Ministry of Health, Kinshasa, Democratic Republic of Congo; 2 World Health Organization, Kinshasa, Democratic Republic of Congo

**Keywords:** Routine Immunization, routine vaccination, urban, reaching every district

## Abstract

**Introduction:**

Vaccination coverage of the first dose of diphtheria-tetanus-pertussis-hepatitis B-*Haemophilus influenza* type b (pentavalent) vaccine for the City-Province of Kinshasain the years 2012 – 2014 wasbelow the national objective of 92%, with coverage less than 80% reported in 12 of the 35 health zones (HZ). The purpose of this study was to discern potential contributing factors to low vaccination coverage in Kinshasa.

**Methods:**

We conducted a multi-stage cluster household study of children 6 – 11 months in households residing in their current neighborhood for at least 3 months in the 12 high risk HZ in Kinshasa. Additional information on vaccination status of the children was collected at the health facility.

**Results:**

Of the 1,513 households with a child 6-11 months old, 81% were eligible and participated. Among the 1224 children surveyed, 96% had received the first dose of pentavalent vaccine; 84% had received the third dose; and 71% had received all recommended vaccines for their age. Longer travel time to get to health facility (p=0.04) and shorter length of residence in the neighborhood (p=0.04) showed significant differences in relation to incomplete vaccination. Forty percent of children received their most recent vaccination in a facility outside of their HZ of residence.

**Conclusion:**

This survey found vaccination coverage in 12 HZs in Kinshasa was higher than estimates derived from administrative reports. The large percentage of children vaccinated outside of their HZ of residence demonstrates the challenge to use of the Reaching Every District strategy in urban areas.

## Introduction

Vaccination has been demonstrated to be a highly effective means to fight disease [1]. In Sub-Saharan Africa, despite the availability of vaccines against many infectious diseases and the efforts from governments and their partners to vaccinate every child in the community where they live, the mortality rate from vaccine-preventable diseases for children under five remains among the highest in the world [2]. In order to reach the full potential of vaccination, the Global Vaccination Action Plan (GVAP) [1] and the regional goals for Africa aim to reach at least 90% vaccination coverage for routinely recommended vaccines at the national level and at least 80% in all the health districts by 2020 [3].

The Democratic Republic of Congo (DRC) provides vaccination free of charge through its public, faith-based and private for-profit clinics. As of 2015, 10 antigens were offered during 5 healthcare encounters from birth through 9 months of age. Since 2003, 5 new vaccines have been introduced; the most recent introduction was Inactivated Polio Vaccine (IPV) in 2015. DRC national vaccination objectives for 2015 include 92% of children under 1 vaccinated with the first dose of diphtheria-tetanus-pertussis-hepatitis B-Haemophilus influenza type b (pentavalent or Penta) and 90% vaccinated with measles containing vaccine (MCV) through routine vaccination services [4].

The City-Province of Kinshasa, the capital of the DRC, has an area of 9,965Km² and an estimated population of 11.6 million inhabitants [5]. A comparison of the first dose of Penta and MCV coverage using 2014 administrative data showed coverage that remained below global and national targets reaching 89% and 79%,respectively. Low vaccination coverage could lead to outbreaks of measles, polio and other vaccine preventable diseases. The mid-term review organized in July 2014 showed 12 (34%) of 35 Health Districts (called Health Zones, HZ) in Kinshasa with a coverage of the first dose of Penta lower than 80%, representing about 20,000 children under one year of age who were expected for the first dose of Pentavalent vaccine and did not receive it (Table 1). Using an earlier survey [6] and field experience, program staff postulated that families were not accessing vaccination services for one or more of the following reasons including that they lacked information of when and where to go; were not reached by community volunteers (French abbreviation: ReCo) or other traditional channels of information; were only recently arrived in the area; found health services unpleasant or time consuming due to crowding; found vaccination cost too much money; or feared side effects. Additionally, children who received their first vaccination were postulated to be failing to complete the primary series for one or more of the following reasons including that families lacked information of when to return; were not accessible for defaulter tracing; or were discouraged because health services were crowded and consumed too much time.

**Table 1:** Target population and coverage of first dose of pentavalent vaccine (Penta1) in 12 high risk Health Zones in Kinshasa, DRC, data Jan-July 2014

Health zone	Target population EPI, 0-11 months	Coverage Penta1	Children 0-11 months not vaccinated, Penta1
Bandalungwa	6,661	73%	899
Barumbu	5,567	46%	1,503
Binza-Ozone	13,718	55%	3,087
Bumbu	15,190	62%	2,886
Kalamu I	5,347	78%	588
Kingasani	8,123	62%	1,543
Kintambo	3,837	59%	787
Lemba	11,805	64%	2,125
Lingwala	3,154	51%	773
Makala	11,274	60%	2,255
Masina I	12,247	61%	2,388
Ngaba	6,670	67%	1,101
Total	103,593	61%	19,935

**Source : D**RCMinistry of Health EPI database**:**January to June 2014

To identify potential reasons why families in these high-risk HZ were not accessing vaccination services or completing the series, we conducted a household survey of the routine vaccination status of children 6 to 11 months of age. Additionally, we explored household and residential neighborhood factors for those children to examine possible differences between those who do and do not receive all the recommended vaccines for one’s age.

## Methods

We conducted a cross-sectional, stratified, 3 stage cluster survey of children 6 – 11 months old (mo) in12 high risk HZs in Kinshasa. The survey methods are described in detail elsewhere [7]. A quota of households in randomly selected neighborhoods were visited and a structured interview was conducted with the parent or caregiver present in the household and most familiar with the child’s vaccinations; the family was included only if they had resided in the selected neighborhood for at least 3 months prior to the interview. and provided verbal consent. In addition to the questions regarding routine vaccination and household characteristics, the respondent was asked about their experiences in the health facility where the child either had received the most recent vaccination or, in case the child had never been vaccinated, where the child had received the most recent curative healthcare. As many of the identified facilities as could be located within the 12 HZ were visited and the vaccination history of the children copied from the immunization register. Vaccination coverage was calculated based on the history provided by the parent, and if available, the information in the vaccination card and/or the vaccination register. A vaccination was considered received if found in any of the three sources with no attempt to reconcile differences. Up to date for vaccination was calculated based on the recommended vaccines for the child according to age. IPV was not included in the definition of up to date as it was introduced in Kinshasa in April 2015, and therefore not recommended for many of the surveyed children.

Data were entered into a CSPro database and analyzed using SAS v.9.4. We calculated the percentages and Wilson confidence intervals accounting for the strata (HZs) and clusters (residential clusters). Confidence intervals were calculated at the 95% level. We tested significance using 2nd order Rao-Scott chi-square tests; p-values less than 0.05 were considered significant. The assessment was classified as a routine public health program evaluation by the human subject coordinator at the Centers for Disease Control and Prevention in the United States. In DRC, the assessment received approval from the national ethics committee.

## Results

Of the 1,513 households with a child 6-11 months old, 19% were ineligible due to residence in the neighborhood less than 3 months, unavailability to be interviewed, or refusal to participate; 81% were eligible and participated. Parents or caregivers responded for 1,224 children (6–11 months) [7].

### Demographics of the study respondents

The demographics of the study respondents are presented in Table 2. Among the children surveyed, 46% were female, 32% were first born, and 96%d were born at the hospital. Twenty seven percent of the households had lived in the neighborhood for the last 3 – 11 months and 94% owned at least one mobile phone. In 56% of the households, 5 – 9 people live in the dwelling. The majority of mothers of the surveyed children were married or cohabitating (79%), completed elementary school (73%), were Christian (93%), and did not work outside of the home (66%).

**Table 2:** Demographic characteristics among households interviewed in 12 high risk HZ in Kinshasa, DRC (n=1224)

	N^+^	%
**Gender**		
Masculine	655	54
Feminine	568	46
**Vaccination data source**		
Home-basedcard	860	74
Facility based register (no card)	104	9
Caregiverrecallonly	206	18
**Birth order of child**		
First	387	32
2-4	665	54
5-9	171	14
**Length of residence in neighborhood**		
3m-11m	315	27
12-23m	248	21
24-59m	342	29
5+yrs	276	23
**Mother’s age**		
<20yrs	101	9
20 – 24 yrs	291	25
25 – 29 yrs	334	28
30 – 34 yrs	243	21
35 – 39 yrs	163	14
40+ yrs	47	4
**Mother’s marital status**		
Married/cohabitation	970	79
Not married	252	21
**Mother’s education**		
None	60	5
Primary	885	73
Secondary	254	21
Post-secondary	6	1
**Mother’s religion**		
Catholic	151	12
Other Christian	989	81
Muslim	15	1
Other	71	6
**Mother’s occupation**		
Works outside the home/student	418	34
None/housewife	803	66
**Household owns mobile telephone**		
Yes	1146	94
No	74	6
**Number of persons in household**		
2-4	342	28
5-9	684	56
10+	194	16

+ Numbers excludemissing data

### Vaccination coverage

Coverage from 18% of children (n=206) was estimated from parental recall only, 74% (n=860) estimated from card, and 9% (n=104) from the facility-based register (Table 2). Vaccination coverage by antigen is presented in Table 3. Excluding doses typically given on the maternity ward, more than 90% of children 6-11 months had at least a first contact with vaccination services (OPV1: 93% (CI 91-95%), Penta: 96% (CI 94-97%), PCV: 95% (CI 93-97%)). Coverage for third doses of vaccines in the national schedule was 77% (CI 74-80%) for OPV, 84% (CI 81-86%) for Penta and 84% (CI 81-86%) for PCV. Eighty five percent (CI 80-88%) of the children 10-11 months old were vaccinated against measles during routine vaccination, and 84% (CI 80-88%) against yellow fever. Overall, 71% (CI 68-75%) of the children were up-to-date for their age at the time of the survey. The vaccination coverage is presented by HZ in Table 4. The vaccination coverage for Penta 1 ranged from 90-99%, and 76%-97% for MCV.

**Table 3:** Vaccination coverage among children 6-11 months old living in 12 high risk Health Zones in Kinshasa, DRC, by antigen+ (N=1224)

	**Recommended age of administration**	**Coverage**		
		**n**	**%**	**95% CI**
BCG	Birth	1196	98	96, 98
OPV0	Birth	1147	94	91, 95
OPV1	6 weeks	1144	93	91, 95
OPV2	10 weeks	1079	88	85, 90
OPV3	14 weeks	962	77	74, 80
Penta1	6 weeks	1168	96	94, 97
Penta2	10 weeks	1142	93	91, 95
Penta3^¥*^	14 weeks	1035	84	81, 87
PCV1	6 weeks	1164	95	93, 97
PCV2	10 weeks	1131	93	91, 94
PCV3	14 weeks	1028	84	81, 86
MCV	9 months	327^++^	85	80, 88
Yellow Fever	9 months	325^++^	84	80, 88
Up-to-date for age		889	71	68, 75

BCG=Bacille Calmette Guerin; OPV= Oral Polio Vaccine ; Penta= Pentavalent (diphtheria-tetanus-pertussis-hepatitis B-Haemophilus influenza type b); PCV=Pneumococcal Conjugate Vaccine ; MCV=Measles Containing Vaccine

+ combined data from immunization card, verbal history, and immunization registry

++ children 10-11 mo

¥+ The estimated intra-class correlation (ICC) for Penta3 among 6-11 month olds (average cluster size = 6.8) was 0.12, and among 12-23 month olds was 0.03 (average cluster size = 4.0)

**Table 4:** Vaccination coverage among children living in 12 high risk Health Zones in Kinshasa, DRC, by zone+

	**Penta 1**		**MCV^++^**	
	**%**	**95%CI**	**%**	**95%CI**
Bandalungwa	96	91, 100	83	69, 97
Barumbu	93	89, 98	79	63, 94
Binza-Ozone	96	91, 100	79	64, 93
Bumbu	97	94, 100	82	72, 92
Kalamu I	93	87, 99	85	74, 97
Kingasani	94	89, 100	86	68, 100
Kintambo	94	89, 99	76	55, 96
Lemba	98	94, 100	86	69, 100
Lingwala	98	95, 100	97	89, 100
Makala	90	80, 100	88	77, 99
Masina I	99	97, 100	93	84, 100
Ngaba	96	92, 100	89	76, 100

+ combined data from immunization card, verbal history, and immunization registry

++ children 10-11 mo

When asked if the child had received all recommended vaccinations, a total of 1092 of the 1224 respondents (76%, CI 74-79%) responded yes (Table 5). Among those 1092, 249 (24%) children in fact lacked at least one antigen for their age. Overall, 40% (CI 37-43%) of children were reported to have visited a facility outside of their HZ of residence for their most recent vaccination or curative services.

**Table 5:** Characteristics of children residing in 12 high risk Health Zones in Kinshasa, DRC, by vaccination status (N=1224)

	**Up-to-date^++^**			**Not Up-to-date**			**p**
	**N^+^**	**%^+^**	**95% CI**	**N^+^**	**%^+^**	**95% CI**	
**Gender**							0.27
Masculine	469	70	65, 74	186	30	26, 34	
Feminine	420	73	68, 78	148	27	22, 32	
**Mother thought child was up-to-date**							*<0.001*
Yes	843	76	72, 79	249	24	21, 28	
No	46	37	27, 48	86	63	52, 73	
**Time to arrive to facility**							*0.04*
0-15 minutes	417	77	71, 81	113	23	19, 29	
16–30 minutes	240	75	69, 80	79	25	20, 30	
>30 minutes	117	66	58, 72	60	34	28, 42	
**Price of last vaccination**							0.75
Free	80	69	58, 78	31	31	21, 42	
1-500FC	294	74	69, 79	98	26	21, 31	
501-2000FC	360	75	70, 79	116	25	21, 30	
>2000FC	126	73	64, 81	42	27	20, 36	
**Length of residence**							0.04
3m-11m	210	65	58, 71	105	35	29, 42	
12-23m	181	71	65, 77	67	29	23, 35	
24-59m	259	75	70, 80	83	25	20, 30	
5+years	206	74	67, 80	70	26	20, 33	

+ Numbers exclude missing data

++ Up to date – child received all antigens recommended for age at the time of the survey

Select characteristics of surveyed children by vaccination status is shown in Table 5. Seventy seven percent (CI 71%-81%) of children whose caregiver responded that they lived 0 – 15 minutes from the health facility were up-to-date for vaccination, versus 66% (CI 58-72%) of those who live more than 30 minutes away (p= 0.04). Of the children who had lived in the current neighborhood for the past 3-11 months, 65% (CI 58-71%) were up-to-date compared to 71% (CI 65-77%), 75% (CI 70-80%) and 74% (CI 67-80%) among those who had lived in the neighborhood 12-23 months, 24-59 months and more than 5 years, respectively (p= 0.04). There was no significant difference based on gender (p= 0.34) or cost for the most recent vaccination session (p= 0.9).

### User perceptions

**Figure 1:**
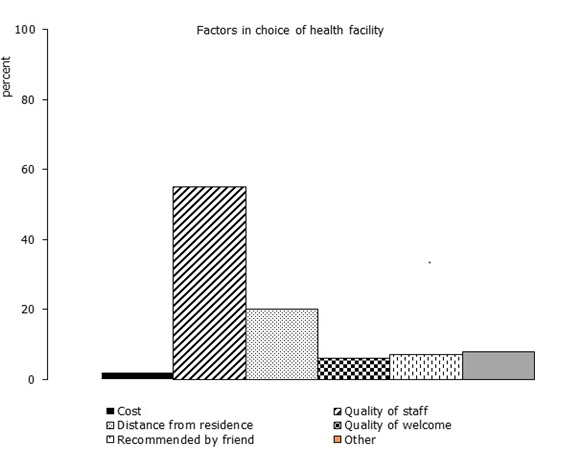
Factor cited by respondent as most important in choice of health facility for most recent vaccination, 12 high risk Health Zones in Kinshasa, DRC

Respondents were asked several questions regarding the facility used most recently for vaccination. The factor cited as the most important in choosing the health facility was quality of staff (56%, CI 52-59%), followed by distance from residence (20%, CI 17-22%) (Figure 1). Regarding the source of information for vaccination (data not shown), 74% of respondents (CI 70%-77%) learned that vaccination was important during antenatal care and/or at delivery at the maternity ward and 74% (CI 70-78%) were told where to get vaccination during antenatal care and/or at delivery at the maternity ward. Few respondents (<2%) reported ReCoas a source of information that vaccination was important or as a location to receive vaccination services.

## Discussion

We found vaccination coverage among children living in twelve high risk HZs in Kinshasa was higher than the reported administrative coverage estimates and that none of the HZs had coverage below 90% for doses recommended at 6 weeks of age, indicating high levels of access to vaccination services. However, only 71% of 6-11 month old children were up to date for their age, indicating in this population more of a problem with children completing the vaccination series. This survey identified significant differences in being up to date for vaccination related to duration of residence in the neighborhood and distance to the health facility. Importantly, almost a quarter of caregivers mistakenly thought that their child was up to date with vaccinations and thus did not seek missing vaccines. This survey did not find price, lack of knowledge about the importance of vaccination or barriers related to the caregiver seeking services as having differences with respect to vaccination status. This survey found that perceived quality of service was the most important factor in caregiver choice of health facility.

Community volunteers (ReCo) are often relied upon by health facilities for communicating with the public regarding vaccination services; however, in our survey these were shown to be the source of vaccination information for only < 2% of respondents. The ReCo’s weak contribution to communication observed in this study is consistent with other studies in urban settings [8]. These factors indicate the need for better communication regarding when and where to receive vaccination through additional channels, while the near universality of hospital births, the relatively higher level of education of the mothers, and the availability of mobile telephones offer opportunities.

Distance to a health facility might indicate the need for additional HF, but in an urban setting, where families have a choice of vaccination sites, determining what factors are considered by caregivers when assessing “quality”, the most cited criteria for choice of vaccination site, and enhancing the services provided in all facilities may create a draw to neighborhood services.

Our survey identified a large discrpenancy between administrative data and actual coverage, 15 to 47 percentage points by HZ for Penta 1 with an average difference of 34% points and median of 31. For the 12 HZs together, the difference is 27 percentage points, a 44% difference between the 2 sources. One reason for this could be that 40% of surveyed children in these 12 HZs were vaccinated outside of their HZ of residence, and thus the dose would have been administratively counted in another HZ. The non-capture of children outside of their HZ of residence may generate errors in the prioritization and planning of activities, for example targeting these HZ for catch up activities though in reality the children are already vaccinated. Although not explored in detail during our survey, another contributing factor to this discrepancy may include poor quality of administrative data which has been demonstrated in other reports [9-11].

These findings raise questions about the effectiveness of the Reaching Every District (RED) strategy, the key vaccine program implementation strategy, in Kinshasa. RED has been extensively evaluated in rural settings [12-16] and relies upon 5 components, 1) planning and management of resources, 2) reaching target populations, 3) linking services with communities, 4) supportive supervision and 5) monitoring for action, for improved vaccination [17]. This study found that families in urban settings have the ability to readily seek services outside of their zone of residence. The effectiveness of RED hinges upon accurate definition of community and catchment population for mobilization, planning and monitoring. The definition of community in this urban context needs to be revisited, new models developed for planning and monitoring vaccination coverage to achieve program objectives.

Survey results were shared with the management teams of the 12 HZs, each of which developed an action plan to address findings in their HZ. Strategies were developed based on evidence from the survey showing that most of the problem with low coverage was due to drop-out, and addressing factors identified by this survey.

This survey had several limitations. The results are limited to an intentional sample of 12 HZs in a large metropolitan area and cannot be considered representative of all children living in Kinshasa. Coverage estimates include verbal history from caregivers which may over or under estimate actual coverage. Other factors, such as distance to and price of vaccination services, relied on recall, which may bias these results.

## Conclusion

In summary, we found vaccination coverage in the 12 HZs in Kinshasa to be higher than program objectives, but failure to complete the primary series remains a serious concern. The high percentage of hospital births, the relatively higher level of education of the mothers and the ubiquity of mobile phones offer opportunities for future interventions to assure that all children complete the vaccination series. This study also challenges the applicability of some components of the RED approach in the 12 HZs in Kinshasa particularly the need for novel approaches to planning, calculating coverage, defaulter tracking and social mobilization in urban environments.

### What is known about this topic

- Distance from the vaccination site limits access to vaccination;
- There is a lack of communication between health workers and mothers on immunization;
- Collateral costs of vaccination (purchase of the pre-school consultation card, payment of the vaccine act) limits access to vaccination.

### What this study adds

- This survey identified significant differences in being up to date for vaccination related to duration of residence in the neighborhood and distance to the health facility;
- The high percentage of hospital births, the relatively higher level of education of the mothers and the ubiquity of mobile phones offer opportunities for future interventions to assure that all children complete the vaccination series;
- This study also challenges the applicability of some components of the RED approach in the 12 HZs in Kinshasa particularly the need for novel approaches to planning, calculating coverage, defaulter tracking and social mobilization in urban environments.

## Competing interests

The authors declare no competing interest.
